# Cardiovalve in mitral valve position—Additional solution for valve replacement

**DOI:** 10.3389/fcvm.2022.960849

**Published:** 2022-09-06

**Authors:** Mohammad Sherif, Simon H. Sündermann, Francesco Maisano, Elisabeth Pieske-Kraigher, Juliane Riess, Markus Reinthaler, Gunther Mai, Tobias Daniel Trippel

**Affiliations:** ^1^Department of Cardiology, Charité – Universitätsmedizin Berlin, Campus Virchow Klinikum, Berlin, Germany; ^2^German Centre for Cardiovascular Research (DZHK), Partner Site Berlin, Berlin, Germany; ^3^Department of Cardiovascular Surgery, Charité – Universitätsmedizin Berlin, Berlin, Germany; ^4^Department of Cardiothoracic- and Vascular Surgery, German Heart Center Berlin, Berlin, Germany; ^5^Department of Cardiac Surgery, Istituto di Ricovero e Cura a Carattere Scientifico (IRCCS) San Raffaele University Hospital, Milan, Italy; ^6^Department of Cardiology, Charité – Universitätsmedizin Berlin, Berlin, Germany; ^7^Department of Cardiac Anesthesia, Charité – Universitätsmedizin Berlin, Berlin, Germany

**Keywords:** mitral insufficiency, heart valvular disease, heart failure, TMVR, comput(eriz)ed tomography

## Abstract

We report on a 72 years old male patient with recurrent heart failure hospitalizations caused by severe mitral regurgitation due to severe restriction of the posterior mitral leaflet treated with the transfemoral mitral valve replacement (TMVR) system Cardiovalve. Immediate interventional success was obtained resulting in a quick mobilization and discharge.

## Introduction

Mitral Regurgitation (MR) is a major risk factor for future morbidity and mortality. Patients with left ventricular (LV) dysfunction who have concurrent MR have a nearly two-fold increased risk of death or hospitalization (1). The current standard is to improve significant MR with surgical mitral valve replacement (MVR) or transcatheter edge-to-edge repair (TEER) (2). Transcatheter mitral valve replacement (TMVR) has emerged as a less invasive approach potentially surmounting some of the current hurdles associated with transcatheter edge-to-edge repair and high-risk mitral valve surgery (3).

## Case report

**Figure 1:**
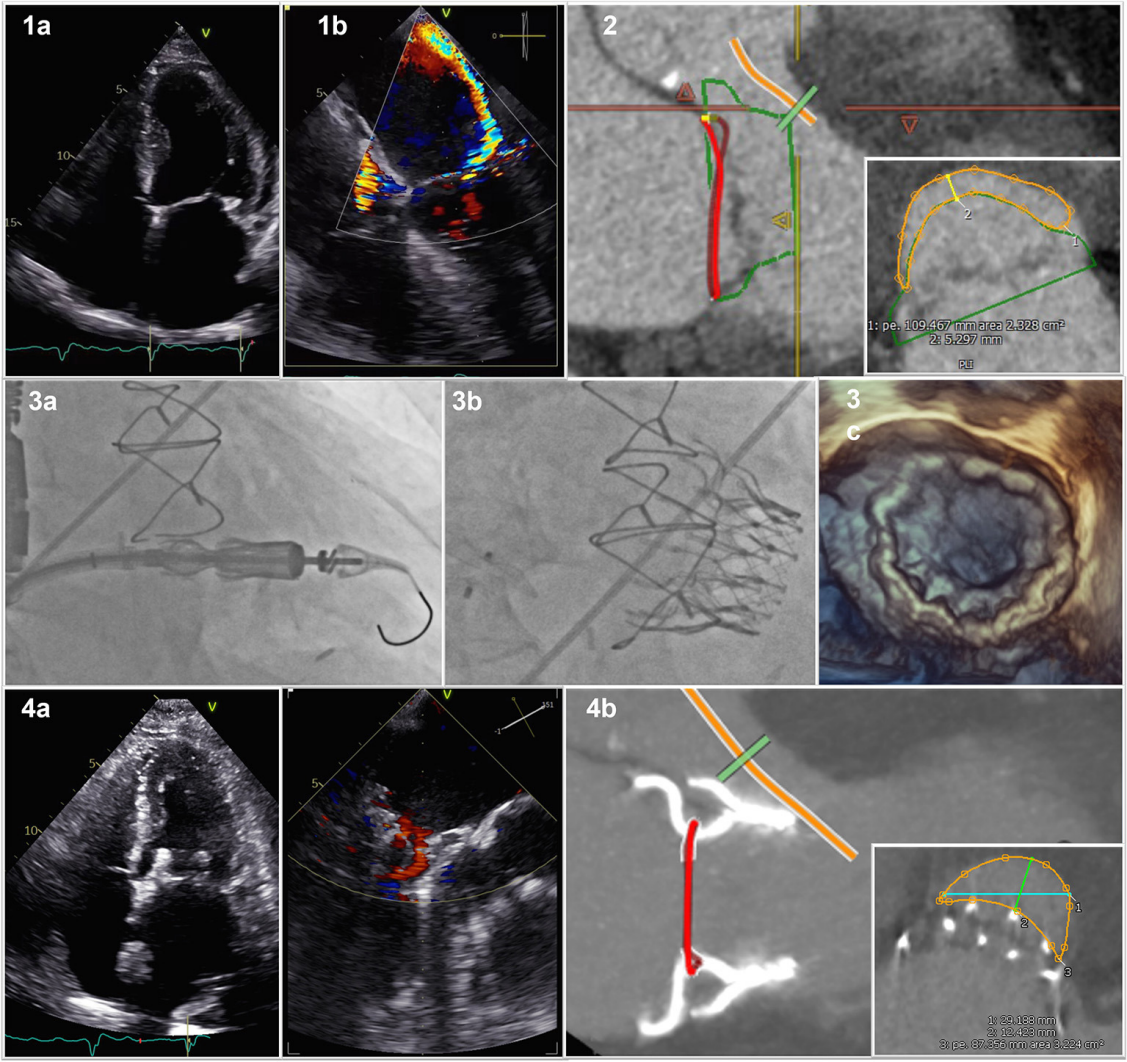
Cardiovalve in Mitral Valve Position. (1a) 2D posterior mitral leaflet by calcification; (1b) consecutive severe excentric posterior jet; (2) Pre-procedural computed tomography scan with valve sizing and predicted neo-LVOT; (3a) Fluoroscopy of device delivery; (3b) Fluoroscopy of device with closure of ASD; (3c) 3D transesophageal echocardiography of delivered Device; (4a) post-interventional result; (4b) Post-procedural CT scan with actual neo-LVOT.

We describe the case of a 72 years old male patient with preserved ejection fraction a recurrent history of decompensated heart failure caused by severe mitral regurgitation due to severe restriction of the posterior mitral leaflet by calcifications (Figure 1,1a) with a consecutive severe excentric posterior jet (Figure 1,1a). The patient has a > 10 years history of coronary heart disease with CABG LIMA/LAD, Vene/RIVP, Vene/D1, and Vene/M1 with preserved left ventricular ejection fraction, a history of stroke, atrial fibrillation, severe chronic obstructive pulmonary disease and pulmonary hypertension as well as chronic kidney disease with a eGFR of 48 ml/min/1.73 m^2^ (by CKD-EPI) resulting in a Euro Score II of 19.40% and STS for Morbidity and Mortality of 33.223% (Version 4.2). Given the clinical condition and patient history he was deemed inoperable for conventional MVR, minimal-invasive right thoracotomy surgical treat treatment or TEER by our institutions interdisciplinary heart team in August 2021.

On March 11th, 2022 he was admitted to our institution with New York Heart Association (NYHA) functional class IV, peripheral edema, pleural effusions and jugular vein distension. His NT-proBNP was 2,894 ng/L and recompensation with intravenous diuretic therapy was initiated. Screening computed tomography scan confirmed anatomic suitability for Cardiovalve prosthesis (Figure 1,2) and projected a neo left ventricular outflow tract (LVOT) of ~2.3 cm^2^. After approval by AHEAD screening committee patient has been enrolled in the AHEAD-Trial (ClinicalTrials.gov, Identifier: NCT03339115) and scheduled for transcatheter mitral valve replacement (TMVR) with the Cardiovalve (Cardiovalve, Or-Yehuda, Israel).

The Cardiovalve is based on a low-profile design of three scallop-shaped bovine pericardial leaflets designed to be delivered through a transfemoral transseptal approach using a 32-F capsule with a 24-F shaft (4). Three valve sizes are available, covering an intracommissural annular size from 36 to 53 mm (4). The patient underwent the procedure on March 16th, 2022 in our hybrid operation theater under guidance with fluoroscopy and transesephageal echocardiography in full anesthesia. A surgical vascular access of the right femoral vein was established. Transeptal puncture was performed employing the VersaCross® Transseptal (Baylis Medical, München, Germany) System under balloon dilatation of the intraatrial septum with a 10 mm balloon. The delivery system was inserted into the left atrium and centered with respect to the mitral annulus before final deployment and stabilized with a 300 cm Lunderquist in over the wire technique under fluoroscopic guidance (Figure 1,3a). We implanted a 45 mm Cardiovalve Mitral Valve replacement in a stepwise procedure. Mean transvalvular gradient was <4 mmHg. The procedure related atrial septum defect was closed using a 12 mm Amplatz Occluder (Abbott, Abbott Park, IL, USA) (Figure 1,3b). Transesophageal echocardiography showed the correct positioning of the valve, with no leak, and no LVOT (Figure 1,3c). The patient was extubated in the hybrid operation theater immediately after the procedure, transferred to our intermediate care ward for 24 h for surveillance and hence transferred to our normal care ward for another 5 days of recompensation and mobilization.

Post-procedural echocardiography showed a satisfactory result (Figure 1,4a), CT scan confirmed prosthesis stability, no evidence of left ventricular outflow tract (LVOT) obstruction (Figure 1,4b) with an actual neo-LVOT of ~3.2 cm^2^ before discharge. We did observe a clinical improvement to NYHA functional class II and rapid recompensation. The patient was discharged on 25th of March 2022 and the 30 day post-discharge timeframe was event free. Given the pre-existing indication for oral anticoagulation due to atrial fibrillation, the patient continued to take warfarin after discharge. The present report shows the feasibility of TMVR with a fully percutaneous antegrade system in the setting of a severe restriction of the posterior mitral leaflet.

## Discussion

The case we report here focuses on a patient with clinical signs and symptoms of heart failure with recurrent heart failure hospitalizations due to significant mitral valve disease. Despite his young age, he was deemed inoperable. Severe leaflet restriction and calcification hindered the option of TEER. The ongoing interventional revolution in the field of structural heart disease brought a novel treatment option to the hybrid operation table: Cardiovalve.

Thanks to the rapidly evolving field, we were able to treat this patient within the European Feasibility Study of the Cardiovalve Transfemoral Mitral Valve System (AHEAD) and obtained fine short term clinical, structural and imaging results. Of special note, the actually obtained neo-LVOT was greater than the predicted neo-LVOT. The low-profile design of three scallop-shaped bovine pericardial leaflets employed in the Cardiovalve enabled a considerate treatment in a patient deemed clinically unfit for conventional MVR as well as anatomically unfit for minimal-invasive right thoracotomy surgical treat treatment or TEER.

Yet, structured outcome data on short and long-term morbidity and mortality remain lacking. Safety, performance and outcome data of TMVR devices such as Cephea®, Evoque®, Intrepid®, Sapien M3®, and NaviGate® competing with Cardiovalve® in some aspects will have to be taken into account.

TMVR will further advance the field of transcatheter treatments of mitral valve disease and allow promising treatment options for many patients.

## Data availability statement

The raw data supporting the conclusions of this article will be made available by the authors, without undue reservation.

## Ethics statement

The studies involving human participants were reviewed and approved by LAGESO, BfArM. The patients/participants provided their written informed consent to participate in this study.

## Author contributions

MS and TT conceptionalized, drafted, and wrote the article. SS, FM, GM, MR, JR, and EP-K reviewed the article for important intellectual content. All participated in patient care. All authors contributed to the article and approved the submitted version.

## Conflict of interest

Author MS reports to be principal investigator of the AHEAD trial at the respective site and received honoraria from Edwards Lifesciences, Abbott and Cardiovalve. Author FM reports honoraria from Edwards Lifesciences, Abbott and Cardiovalve. The remaining authors declare that the research was conducted in the absence of any commercial or financial relationships that could be construed as a potential conflict of interest.

## Publisher's note

All claims expressed in this article are solely those of the authors and do not necessarily represent those of their affiliated organizations, or those of the publisher, the editors and the reviewers. Any product that may be evaluated in this article, or claim that may be made by its manufacturer, is not guaranteed or endorsed by the publisher.
